# Rationale for and protocol of a multi-national population-based bacteremia surveillance collaborative

**DOI:** 10.1186/1756-0500-2-146

**Published:** 2009-07-22

**Authors:** Kevin B Laupland, Henrik C Schønheyder, Karina J Kennedy, Outi Lyytikäinen, Louis Valiquette, John Galbraith, Peter Collignon, Deirdre L Church, Daniel B Gregson, Pamela Kibsey

**Affiliations:** 1 Departments of Medicine and Pathology and Laboratory Medicine, University of Calgary and Calgary Laboratory Services, Calgary, Alberta, Canada; 2 Department of Clinical Microbiology, Aalborg Hospital, Aarhus University Hospital, Aalborg, Denmark; 3 Infectious Diseases Unit and Microbiology Department, The Canberra Hospital and School of Clinical Medicine, Australian National University, Woden, Australian Capital Territory, Australia; 4 Department of Infectious Disease Epidemiology, Hospital Infection Program, National Public Health Institute, Helsinki, Finland; 5 Department of Microbiology-Infectious Diseases, Université de Sherbrooke, Sherbrooke, Québec, Canada; 6 Microbiology Laboratory, Vancouver Island Health Authority, Royal Jubilee Hospital, Victoria, British Columbia, Canada

## Abstract

**Background:**

Bloodstream infections are frequent causes of human illness and cause major morbidity and death. In order to best define the epidemiology of these infections and to track changes in occurrence, adverse outcome, and resistance rates over time, population based methodologies are optimal. However, few population-based surveillance systems exist worldwide, and because of differences in methodology inter-regional comparisons are limited. In this report we describe the rationale and propose first practical steps for developing an international collaborative approach to the epidemiologic study and surveillance for bacteremia.

**Findings:**

The founding collaborative participants represent six regions in four countries in three continents with a combined annual surveillance population of more than 8 million residents.

**Conclusion:**

Future studies from this collaborative should lead to a better understanding of the epidemiology of bloodstream infections.

## Introduction

Bloodstream infections are among the most important causes of death in developed countries and cause significant morbidity and healthcare cost [1-5]. Bloodstream infections may arise in community-based patients, or may complicate patients course admitted to hospital as nosocomial infections. Population-based studies conducted in Denmark (1981–1994), Finland (1995–2002), and the United States (2003–2005) have reported overall incidence rates ranging from 76 to 189 per 100,000/year with the highest rates observed in the most recent years [2,4,5]. One study from Canada reported that community-onset bloodstream had a similar acute burden of disease as each of major trauma, stroke, and myocardial infarction [1].

Population-based surveillance has been recognized as an optimal means to define burden of disease, evaluate risk factors for acquiring infections, and for monitoring temporal trends in occurrence and resistance. Because all episodes of disease occurring in a defined population at risk are included in these designs, selection bias is minimized and calculation of incidence and mortality rates are facilitated [6]. Several regions worldwide have reported on population-based bloodstream infection surveillance data or systems including Australia [7,8], Canada [1,9-11], Denmark [12-15], Iceland [16,17], Finland [4,18-20], and the United States [2]. National programs with high coverage for selected blood culture pathogens are also operative in a number of European countries [21-24]. However, to date approaches between intercontinental regions have not been coordinated.

A coordinated multi-national effort would have numerous potential benefits. First, inter-regional (national, continental) differences in incidence, risk factors, outcomes, and resistance rates may be directly compared. Second, a surveillance region spanning several countries in different continents that includes a large (millions per year) surveillance population would allow the study of rare isolates and facilitate the early detection of emerging organisms. Finally, the recognition of the importance of globalization of trade and international travel in the spread of multi-resistant bacterial pathogens including extended spectrum β-lactamase producing *Escherichia coli *and *Salmonella typhimurium *DT104 has highlighted that infectious diseases control is a global concern [25,26]. While initially this proposed collaborative will include a few selected regions within developed countries, the ultimate goal will be broad multi-national participation.

## Methods and design

There are two requirements for centers to participate in the collaborative: 1) the area of surveillance must be geographically and demographically (age and gender distribution known or estimated) definable and 2) all (≥90%) positive blood cultures occurring among residents of the surveillance population must be identified. Infections identified but occurring in non-residents, and duplicate specimens from residents must be able to be excluded. For common blood culture contaminants, two positive cultures within a five day period will be required for inclusion.

While these features define a minimum criteria set for involvement, it is also preferable, but not mandatory for detailed microbiology results including antimicrobial susceptibility testing of isolates to be available. In addition, detailed clinical information including co-morbidity data, treatments rendered, and hospitalization duration and outcome will be sought where feasible. It is desirable that cases be definable as to location of onset, either as nosocomial [first occurred >48 h (or 2 days) after hospital admission or within 48 hours (2 days) of hospital discharge] or community onset [first identified in community or within 48 hours (2 days) of hospital admission]. There are currently six centres in four countries in three continents presently participating in the developing collaborative. These centres are summarized in Table 1 and each is described in further detail the following sections.

**Table 1:** Characteristics of the proposed participating sites

Region	Population in 2007	Estimated ascertainment	Source of laboratory surveillance	Clinical and outcome data	Start of independent routine surveillance
Calgary Health Region, Canada	1.24 million	≥99%	Regional microbiology laboratory	Routinely available	2000

Canberra Region, Australia	380,000	≥95%	Two public and one private laboratories	Routinely available	1998

Finland	5.30 million	100%	National Infectious Disease Register	Possible though database linkage	1995

North Denmark Region, Denmark	580,000	100%	Regional microbiology laboratory	Routinely available	1981

Sherbrooke, Canada	152,000	≥99%	Regional microbiology laboratory	Routinely available	1999

Vancouver Island Health Authority, Canada	746,000	≥99%	Regional microbiology laboratory	Possible though database linkage	2008

### Calgary Health Region, Calgary, Alberta, Canada

The Calgary Health Region (CHR) provides virtually all acute medical and surgical care to the residents of the cities of Calgary and Airdrie and a large surrounding area (37,000 km^2^; population 1.24 million) in the Province of Alberta, Canada through the publicly funded health system. The region is geographically well defined and isolated from other major centers. Acute inpatient care is provided principally through four major hospitals that have more than 2,000 beds representing 95% of the bed capacity in the region. One regional laboratory system (Calgary Laboratory Services) performs ≈99% of all blood culture testing from both community and hospitals in the CHR. Since 2000 routine ongoing population-based laboratory surveillance for all blood isolates has been undertaken. A database has been developed that includes detailed clinical and outcome data on all admitted bacteremic patients.

### Canberra Region, Australia

The Canberra Region (TCR; population 380,000) includes the city of Canberra within the Australian Capital Territory and the satellite city of Queanbeyan and several small surrounding rural towns within the state of New South Wales. The region is geographically well-defined and isolated, with the nearest city Goulburn (population 21,000) and tertiary care hospitals (Sydney), 100 and 300 kilometres away respectively. TCR is serviced by three public hospitals (730 beds combined) and three private hospitals (335 beds combined). Three of the four microbiology laboratories in TCR are included in routine surveillance and are estimated to capture more than 95% of all positive blood cultures in TCR. The one non-participating laboratory is a private laboratory based in Sydney that only provides non-acute outpatient services and rarely has positive blood cultures. Basic demographic data (age, gender) and residency status based on residential postcodes are available for all cases, and since 1998 further clinical and outcome data has been routinely available for all positive blood cultures involving emergency department assessments or hospital admissions within the two major public hospitals.

### Sherbrooke, Quebec, Canada

Sherbrooke (population 152,000) is the capital of Estrie, a predominantly rural region located in southeastern Quebec, Canada [11]. Sherbrooke is served by a single microbiology laboratory located in the Centre Hospitalier Universitaire de Sherbrooke, a 686-bed academic, tertiary care centre. The population of the Estrie region is geographically captive, since no other major hospitals are located within an 80 km radius. Consequently, an ascertainment rate approaching 100% is estimated for surveillance of bacteremias occurring among regional residents. A clinical data warehouse articulated around a computerized patient data system has been functional since 1999. Detailed demographic, clinical, microbiological, and hospital outcome data is therefore available.

### National Infectious Disease Register (NIDR), Helsinki, Finland

Finland has a population of 5.3 million residents. All acute care is administered through the publicly funded National Health Care System. All Finnish clinical microbiology laboratories report all bacterial isolations from blood to the National Infectious Disease Register (NIDR). With each notification, the following information is transmitted: date and type of specimen, date of birth, gender, and place of treatment and since 2004 each individual's National Identity Code by which the dates of deaths can be obtained from the national Population Information System. Detailed clinical data on all the hospitalizations of patients are available from the National Hospital Discharge Registry (HILMO). This registry contains comprehensive health care records from all hospitals and municipal health centers, including outpatient surgery. While these detailed clinical data from the HILMO are available, they are not routinely maintained as linked data to bacteremias within the NIDR database.

### North Denmark Region, Denmark

The North Denmark Region is located in the northernmost part of the Jutland peninsula (8,000 km^2^; population 580,000). The main city is Aalborg (population 195,000). The region was established by an administrative reform effective 1 January 2007 and the core area is the former North Jutland County (6,000 square kilometers, population 495,000). Comprehensive hospital care is provided through 4 publicly funded hospitals at 11 different sites with a total of 1700 beds. Aalborg Hospital serves the city of Aalborg and is the regions' referral and teaching hospital; the other hospitals are community-based and some facilities offer specialized care only. Bacteriological services for the entire region are provided through the Department of Clinical Microbiology at Aalborg Hospital. Since 1981 all episodes of bacteremia have been registered in the North Jutland Bacteremia Research Database. Detailed clinical and outcome data are available through linkage between relevant administrative and health registries databases including the Danish Civil Registration System and the Hospital Discharge Registry of North Jutland [27].

### Vancouver Island Health Authority (VIHA), British Columbia, Canada

The VIHA includes all of Vancouver Island and an adjacent area of the mainland of British Columbia, Canada. The region (total population 746,000) is comprised by three local health areas including the south (364,000) that includes the provincial capital city of Victoria, the central (262,000), and the north (120,000) covering a total area of more than 56,000 square kilometres. VIHA provides a broad range of community and hospital based medical and surgical services through the publicly funded health system. Inpatient care is provided through 15 facilities representing a total acute care capacity of 1,500 beds. Blood culture testing for all hospitals and the majority of outpatients in VIHA is administered by the regional microbiology laboratory. One private laboratory performs outpatient blood cultures but this represents an estimated <1% of all positive blood cultures in the region. Although not routinely available in the microbiology database, an advanced information technology infrastructure exists whereby detailed clinical and outcome data may be obtained on all admissions to the 15 acute care centres in VIHA.

## Discussion

This multinational surveillance co-operative aims to develop a better understanding of the epidemiology of bloodstream infections in several developed countries in three continents. Because of the collaborative power from a large combined surveillance area, the study of rare isolates and patient groups will be enabled. For example, *Fusobacterium necrophorum *is an uncommon but important cause of bacteremia and is the principal causative agent of Lemierre syndrome. Much of what is known about this organism is based on compilations of anecdotal case reports, and a single population-based study of 24 cases (incidence approximately 1.5 per million/year) from Denmark [28]. Based on an incidence rate of 1–2 per million per year, our collaborative would potentially be able to systematically identify 100–200 incident cases since 2000 based on our combined surveillance population of more than 8 million residents per year. Another advantage of our collaborative is that because the surveillance population will represent a broad range of geographical regions, with mixtures of urban and rural residents with a range of socioeconomic and demographic differences, studies investigating the determinants of disease will be facilitated. For example, non-typhoidal *Salmonella *species infections are principally related to animal and contaminated food exposures [29]. If we observed differences in incidence among regions, then ecological associations between urbanicity, agricultural practices, and food sources may be further explored. A final major advantage is in the assessment of preventive interventions at the population level such as with vaccination. This has been exemplified by the introduction of universal 7-valent pneumococcal conjugate vaccine in populations with a subsequent reduction in both the target childhood populations as well as adults. While several countries have independently identified such an effect [30], our collaborative would allow such an evaluation but with the advantage of concurrent control populations where interventions are introduced at different time periods among regions.

There are a number of potential challenges. While in many cases centre-based surveillance is currently supported through routinely funded programs, in others resources have not been allocated and at present no specific funds have been allocated. Principal expenses include support for database management, data analysis, and for investigator meetings either directly or through teleconference. A second major consideration is with the sharing of data among the collaborative. In part because of both patient confidentiality and intellectual property reasons, a central collaborative database is not proposed. Rather, each centre will autonomously maintain their own database utilizing the collaborative definitions and will generate standardized summary reports. Each centre will require compliance with the specific regulations for scientific and ethical approval and data protection. Finally, we are aware of the limitation that the collaborative currently only includes six regions, all in developed countries. The collaborative openly welcomes new participants that are capable of providing the specified bacteriological and demographic information. Regions in South America, Africa and Asia would particularly valuable additions.

## Competing interests

The authors declare that they have no competing interests.

## Authors' contributions

This article was drafted by KL. All authors contributed to information collection and critical revision and approval of the manuscript.
